# Current progress in targeted pharmacotherapy to treat symptoms of major depressive disorder: moving from broad-spectrum treatments to precision psychiatry

**DOI:** 10.1017/S1092852925000094

**Published:** 2025-02-07

**Authors:** Manpreet K. Singh, Michael E. Thase

**Affiliations:** 1 Department of Psychiatry and Behavioral Sciences, University of California Davis Health, Sacramento, CA, USA; 2 Perelman School of Medicine and Corporal Michael J Crescenz Veterans Affairs Medical Center, University of Pennsylvania, Philadelphia, PA, USA

**Keywords:** psychiatry, precision psychiatry, major depressive disorder, MDD, pharmacotherapy

## Abstract

Major depressive disorder (MDD) is a disabling condition affecting children, adolescents, and adults worldwide. A high proportion of patients do not respond to one or more pharmacological treatments and are said to have treatment-resistant or difficult-to-treat depression. Inadequate response to current treatments could be due to medication nonadherence, inter-individual variability in treatment response, misdiagnosis, diminished confidence in treatment after many trials, or lack of selectivity. Demonstrating an adequate response in the clinical trial setting is also challenging. Patients with depression may experience non-specific treatment effects when receiving placebo in clinical trials, which may contribute to inadequate response. Studies have attempted to reduce the placebo response rates using adaptive designs such as sequential parallel comparison design. Despite some of these innovations in study design, there remains an unmet need to develop more targeted therapeutics, possibly through precision psychiatry-based approaches to reduce the number of treatment failures and improve remission rates. Examples of precision psychiatry approaches include pharmacogenetic testing, neuroimaging, and machine learning. These approaches have identified neural circuit biotypes of MDD that may improve precision if they can be feasibly bridged to real-world clinical practice. Clinical biomarkers that can effectively predict response to treatment based on individual phenotypes are needed. This review examines why current treatment approaches for MDD often fail and discusses potential benefits and challenges of a more targeted approach, and suggested approaches for clinical studies, which may improve remission rates and reduce the risk of relapse, leading to better functioning in patients with depression.

## Introduction

The World Health Organization describes depression as a leading cause of disability, with an estimated 280 million individuals affected worldwide.^1^ Although effective treatments for depression exist, more than 25% of patients with major depressive disorder (MDD) do not respond to 2 or more treatments.^2^ Further, the onset of benefits of antidepressants can be slow,^3^ and guidelines now suggest that it takes up to 12 weeks of treatment to ensure an optimal treatment response.^4^ ^,^^5^

MDD treatments have evolved significantly over the past 60 years,^6^ with therapies becoming increasingly targeted or selective. Before the mid-1950s, the only effective medical treatment for severe depressive episodes was electroconvulsive therapy.^7^ Iproniazid, a medication used to treat tuberculosis, was the first drug identified with antidepressive effects; within a few years, its mechanism of action was linked to the inhibition of monoamine oxidase.^3^ Monoamine oxidase inhibitors (MAOIs) do not act on specific receptors but increase the levels of serotonin, norepinephrine, and dopamine in the brain by preventing their enzymatic oxidation.^3^ ^,^^8^ Shortly thereafter, the therapeutic effects of imipramine, the first drug to be classified as a tricyclic antidepressant (TCA), were identified in the course of research to develop safer and more effective antipsychotic drugs than chlorpromazine.^3^ TCAs inhibit presynaptic norepinephrine and, to a lesser extent, serotonin reuptake transporters.^3^

Following the goal to develop interventions with fewer side effects than these serendipitously discovered medications, selective serotonin reuptake inhibitors (SSRIs) became the most commercially successful class of antidepressant drugs following the introduction of fluoxetine in 1987.^8^ ^,^^9^ Though reduced, side effects associated with SSRIs were still problematic for some patients, and others did not achieve meaningful symptomatic improvement, leading to the development of antidepressants such as bupropion, venlafaxine, reboxetine, and mirtazapine.^8^ ^,^^10^ Although these medications offered additional options for patients, none were able to supplant SSRIs as the standard first choice for first-line therapy. Moreover, as was the case with the TCAs and MAOIs, there was often substantial latency between beginning therapy and the onset of meaningful clinical benefits.^3^ ^,^^8^ Moreover, despite having certain advantages in tolerability and safety indices, the so-called second generation of antidepressants was not more effective than the TCAs or MAOIs.^8^ Some suggested that the unmet needs in the pharmacotherapy of depression, such as the long latency to response and an apparent plateau in effectiveness across classes of antidepressants, were attributable to the fact that all these medications targeted monoaminergic mechanisms.^8^ It was further posited that novel targets for pharmacotherapy would need to be identified in order for the next generation of antidepressants to emerge.^8^

By the late 1990s, interest in the glutamatergic system and its importance in the neurobiology of depression had grown. It was recognized that a single intravenous dose of the anesthetic drug ketamine, which blocks the effects of glutamate on the *N*-methyl-D-aspartate receptor, could have rapid and large antidepressant effects.^8^ ^,^^11^ The discovery that the antidepressant effects of ketamine last for a number of days had a transformative effect on depression treatment research, including the commercial development of one of its stereoisomers, esketamine, for intranasal administration.^8^ ^,^^11^ ^,^^12^ However, ketamine and esketamine are classified as controlled substances that can cause dissociation and cardiovascular side effects that warrant up to 2 hours’ of monitoring, which limits their potential for widescale clinical use.^8^ ^,^^13^ ^,^^14^ Nevertheless, the recognition of one novel target for pharmacotherapy that yielded a potentially large and rapid effect for depressed patients helped to restore therapeutic optimism that potentially better options for our patients were on the horizon.^8^ ^,^^11^ ^,^^12^

In the mid-1990s, as it became apparent that the drugs available were not “one size fits all,” a strategy for managing treatment-resistant MDD using an algorithmic approach began to emerge.^15^ This approach, coupled with a systematized monitoring of symptoms and side effects known as measurement-based care, served as the platform for a large-scale study: Sequenced Treatment Alternatives to Relieve Depression (STAR*D).^16^ ^,^^17^ STAR*D comprised a 4-level treatment algorithm in which a patient with depression moved from 1 treatment level to the next, starting with citalopram at level 1 and escalating through levels 2 to 4, which included various switching and combination categories if full remission was not achieved.^17^

In clinical practice, combination treatments are used by many patients to combat treatment resistance and comorbidity.^18^ Results from a meta-analysis indicate that combined treatment results in small-to-moderate improvements in depression compared with psychotherapy or pharmacotherapy alone or with psychotherapy plus a placebo pill.^18^ However, many treatments with different mechanisms of action have been found to have significant adjunctive antidepressant effects.^18^ ^,^^19^ There is little guidance available on the use of one adjunctive therapy over another; new studies are needed to operationalize our understanding of the combination effect.^19^ ^,^^20^

Although continuation and maintenance treatment is generally recommended after a successful response to acute treatment, it is unclear how long maintenance therapy should continue to prevent subsequent recurrent depressive episodes. A measurement-based care approach could enable the monitoring of potential relapse-preventative or disease-modifying effects that have eluded the current treatment armamentarium.^16^ ^,^^21^

The development of pharmacotherapy for MDD has evolved from chance findings to a more targeted neurobiological approach.^8^ A range of specific and targeted therapies are now available; however, there is no objective guidance on how to choose from the many available medications.^22^ Moreover, despite our understanding of the pathophysiology of MDD evolving from single brain region or monoamine deficits to more network-based models with corresponding subtyping,^23^ treatments are generally not targeted to individual phenotypes.

The purpose of this review is to examine why the current approach to MDD often results in treatment failure, the impact of placebo response in clinical trials for MDD, and why more targeted pharmacotherapy for MDD, such as through precision psychiatry, may be beneficial for short-term optimization toward an early treatment response, and in the long-term to reduce the number of trials and ineffective courses of therapy to achieve remission. Further, we will discuss the role of precision psychiatry and how it can be used to inform phenotypes for more targeted treatment and provide suggested approaches for future clinical studies. See Table 1 for a summary of the key points discussed.

**Table 1.** Key points

MDD treatment often results in treatment failure, potentially due to medication nonadherence, a wide range of side effects, inter-individual variability in treatment response, and misdiagnosis.
High placebo response rates in clinical trials delay the development of new antidepressants.
Current treatment guidelines provide general guidance for treatment selection but do not provide a patient-specific approach.
Precision psychiatry, based on neurobiological mechanisms, may offer patients a more tailored treatment approach.
The future of precision psychiatry may be guided through various channels including the further development and refinement of RDoC and other conceptual frameworks that aim to improve detection of biomarkers or by implementation of artificial intelligence to predict best fit treatment options.

## Reasons for treatment failure in MDD

As highlighted above, over one-quarter of patients do not respond to 2 or more treatments and are categorized as having treatment-resistant depression.^2^ Some patients continue to be significantly burdened by depression despite usual treatment efforts and are classified as having difficult-to-treat depression.^24^ It is important to note that while the Diagnostic and Statistical Manual of Mental Disorders, Fifth Edition (DSM-5) provides specific criteria for the diagnosis of MDD,^25^ no such criteria exist for difficult-to-treat depression. While several factors, such as symptom onset time and severity, early treatment response, psychiatric comorbidities, frontal electroencephalography theta activity, neuroimaging, and peripheral markers, have been identified as predictors of antidepressant response,^26^ the rate of nonresponse to antidepressants is still high.^27^ As such, it is important to first understand the possible reasons for treatment failure.^28^ ^,^^29^

Medication nonadherence is an endemic problem that commonly contributes to the apparent “failure” or cessation of the effect of a course of antidepressant therapy after an initial response.^30^ The rates of nonadherence at 4-month follow-up among older adults ranged from 29% to 40% in the USA.^31^ ^,^^32^ In another study examining nonadherence rates in primary care and psychiatric populations across different countries, about 50% of patients were found to discontinue antidepressant medications prematurely.^33^ A systematic review of 21 studies indicated that patient factors (e.g., forgetfulness, comorbidities, and misconceptions about the disease), medication factors (e.g., polypharmacy, side effects, and pill burden), healthcare system-related factors (e.g., physician-patient interactions) and sociocultural factors contributed to the antidepressant nonadherence in patients with MDD.^30^ For all of these reasons, assessment of the history of all patients with difficult-to-treat depression should begin with a careful consideration of adherence.

**Figure 1.**
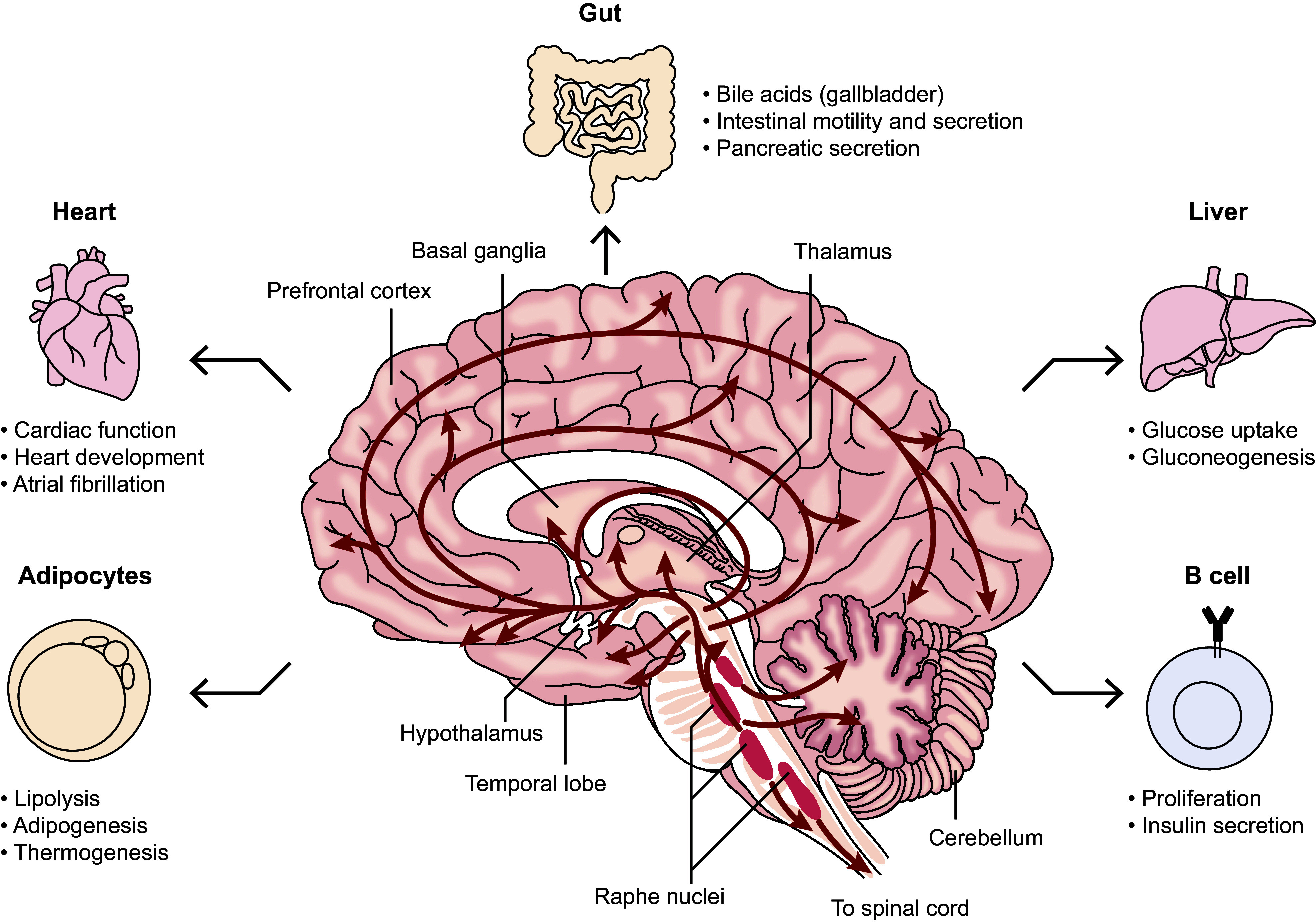
Serotonin neurons target multiple brain structures and other organs, tissues, and cells.

Another potential reason for treatment failure is the “blunt instrument” nature of antidepressants: even relatively selective antidepressants act on many receptors in the brain, often with unwanted effects in the periphery.^28^ ^,^^34^ For example, SSRIs are presumed to work by improving the function of serotoninergic neurotransmission in the brain^29^; however, serotonin is linked to the regulation of not only emotion, mood, stress, appetite, and sleep but also the control of vascular resistance and blood pressure, heart function, mammary gland development, and digestion.^29^ ^,^^35^ SSRIs lead to remission in 30% of patients^29^ ^,^^36^ but are associated with a wide range of side effects, such as memory impairment, somnolence, decreased concentration, fatigue, weight gain, headache, sexual dysfunction, and dizziness.^37^ ^,^^38^ As individual serotonin neurons are highly branched, sending input to multiple forebrain structures (Figure 1), the global targeting of serotonin by SSRIs likely activates antagonistic pathways that may contribute to the side effects.^29^ These unwelcome effects may impact the tolerability and acceptability of SSRIs and may increase the likelihood of medication nonadherence.^33^ Individual serotonin neurons are highly branched and send input to multiple forebrain structures, the midbrain, and the hindbrain (cerebellum). Hence, they target the entire central nervous system^39^; serotonin also targets other tissues and cells.^40^ ^,^^41^

Treatment failure may also be related to the inter-individual variability in treatment response, which has been shown to be heritable and so, in part, is affected by genetic variation.^42^ In a sample of 2799 patients treated with antidepressants, 42% of individual differences in antidepressant response were explained by genetic variants,^43^ which are likely acting together to express a range of behavioral and somatic traits.^44^ A previous study in a Chinese population identified single nucleotide polymorphisms that resulted in poorer treatment responses to fluoxetine and venlafaxine.^45^ However, several genome-wide association studies have not been able to identify genetic associations to robustly predict antidepressant response to date,^42^ ^,^^46^ ^-^^48^ with extant studies either reporting trivial variance explained by genetics or potentially overestimating, due to sample size, the genetic contributions to antidepressant response through the use of genome-wide complex traits or similar analyses.^43^ ^,^^49^ ^,^^50^

Misdiagnosis can also lead to treatment failure and may occur for a variety of reasons, including comorbid disorders and the heterogeneous nature of depressive disorders.^51^ The problem can be compounded by an incomplete understanding of the patient’s condition, resulting in an incomplete or superficial clinical assessment,^52^ leading to a failure to differentiate symptoms of unipolar (i.e., recurrent episodes of MDD) and bipolar depression (BD),^51^ or identifying and addressing mixed depressive states in a person who has never suffered a discrete hypomanic or manic episode.^53^ ^,^^54^ For example, the DSM-5 definition of mixed depression combines manic and depressive symptoms only where the symptoms do not overlap, thereby excluding psychomotor agitation, irritability, and distractibility, which are common symptoms experienced during mixed states.^53^ This can lead to an improper diagnosis and treatment, which may affect the patient’s outcome.

## Case illustration for a patient with mixed features in bipolar depression

A patient with bipolar I depression presented with mixed features for several months with uncontrollable panic, emotional instability, and symptoms of inattention. The patient also had comorbid anxiety and attention deficit hyperactivity disorder (ADHD) and was being treated with lithium (1500 mg/day) monotherapy. Historically, the patient found only modest benefit from combination treatments with quetiapine, gabapentin, SSRI, and stimulants for 12 months and experienced inadequate response. However, when lithium was increased by 300 mg to 1800 mg, the patient experienced further resolution of depressive symptoms, including agitation.

This case suggests that when patients with bipolar I depression with comorbid anxiety and ADHD experience breakthrough mixed feature symptoms, optimizing mood stabilization through dose-finding and adjustment of current medications before treating anxiety and/or ADHD could be a useful first step and mitigate unhelpful polypharmacy. A careful history and stepped decision-making can impact the treatment outcomes for patients who are difficult to treat.

## The impact of placebo response in clinical trials for MDD; adapting trial design

Treatments for depression have both specific and non-specific effects.^55^ In clinical trials, the impact of the non-specific elements of treatment is estimated for the sample by the placebo response rate. However, at the level of the individual, it is usually not possible to separate the specific and non-specific effects of treatment.^56^ Placebo response rates have been documented in hundreds of clinical trials of MDD,^57^ ^,^^58^ with some evidence that the non-specific component of treatment response has increased over the past 30 years.^59^ There is concern that the problem of increasing placebo response has been particularly problematic for investigators studying pharmacotherapy of MDD in children and adolescents.^60^ Factors associated with variation between studies in placebo response rates may include study intervals, the diagnosis criteria used or rater biases in judging depression, baseline severity, trial length, and number of study sites.^57^ ^,^^58^ ^,^^60^ ^,^^61^

High placebo response rates in clinical trials contribute to trial failures and delay the development of new antidepressants.^62^ ^–^^64^ Limiting the number of trial sites, enrolling patients with higher baseline severity at study entry, and implementing protections against expectancy have helped to curb the growth in placebo response rates.^55^ ^,^^61^ Across the past few decades, patients with MDD were likely to benefit from an antidepressant drug by 15% beyond a placebo effect.^61^ Therefore, to adequately address the negative impact of a high placebo response on signal detection, the field needs a better understanding of the developmental, behavioral, social, and biological underpinnings of the placebo response and to effectively model prevailing mechanisms that drive study dropout when placebo is used in clinical trials.^65^

Neuroimaging using positron emission tomography (PET) has shown the changes in the brain due to placebo treatment in a 2-week single-blind, randomized lead-in of 2 identical oral placebos, followed by 10 weeks of open-label treatment.^66^ The oral placebos were described to participants either as being a fast-acting antidepressant agent (active) or disclosed to be an inactive placebo (inactive). When compared with the inactive placebo, clinical responses to the “active” placebo treatment were associated with increased placebo-induced μ-opioid neurotransmission in the subgenual anterior cingulate cortex, nucleus accumbens, midline thalamus, and amygdala.^66^ These results indicate that the variability in patient expectancy likely plays a role in placebo response, and design manipulations that inhibit placebo responses could help separate drug-specific treatment effects in clinical trials.^55^

Furthermore, post hoc examination of clinical trial databases revealed that early improvement or lack of response in the first 2 weeks of blinded therapy is a powerful predictor of subsequent response or nonresponse after 6 weeks of therapy.^67^ ^,^^68^ Strategies such as the sequential parallel comparison design (SPCD) attempt to capitalize on these observations to reduce the placebo response and to increase the efficiency of signal detection in clinical trials.^69^ ^–^^71^ SPCD is a two-stage study design in which a much higher proportion of patients are randomized to receive a double-blind placebo in the first stage.^70^ ^,^^71^ At the end of stage 1, patients from the placebo group are classified as placebo responders or nonresponders; the latter are then re-randomized in a blinded fashion to active drug or placebo in stage 2.^70^ ^,^^71^ However, regulatory agencies such as the Food and Drug Administration (FDA) have not determined if studies using the SPCD method are more likely to succeed than studies using more conventional designs.

## Potential benefits of a more targeted approach

A systematic review and meta-analysis of 522 double-blind studies found that of the 21 antidepressant drugs studied, all were more effective than placebo in adults with MDD.^72^ This suggests that we already have effective antidepressants if treatment is based on neurobiology, neuronal networks of depression, and precision pharmacology, with its focus on diagnosis-based science, not symptoms.^28^ ^,^^34^ However, clinical guidelines are often limited, as they give general information about drug classes and guidance for treatment selection but do not provide further details for the individual compounds.^22^ ^,^^73^ ^,^^74^ There is poor guidance in prescribing guidelines about the possible strategies to personalize antidepressant prescriptions.^22^ ^,^^73^ ^,^^75^ Thus, the choice of an effective antidepressant treatment from over 40 available compounds is still a challenge, as prescription is often based on the personal experience of the clinician.^22^ ^,^^73^

The identification of robust clinical criteria and biomarkers (e.g., neuroimaging biotypes, genetic variants) for guiding both a mechanistic understanding of the disease and treatment choice is important in depression.^76^ Due, in part, to the practical challenges of deep phenotyping with serum and neuroimaging tools with unknown or variable degrees of reliability and validity,^77^ ^,^^78^ consideration should also be given to factors such as past response to antidepressant medication, family pharmacological history, pharmacogenomics to optimize tolerability, and possible drug interactions, which can change medication plasma levels and pharmacodynamics.^22^ ^,^^28^ ^,^^73^ This is especially important given the known associations between depression and health comorbidities such as inflammation and cardiometabolic disease risk.^79^ Considering these factors may lead to more targeted therapy as the right treatments can be matched to the right patients, thereby increasing the benefit–risk ratio.^28^ Personalized treatments could also improve remission rates and reduce the risk of relapse, leading to recovery and better functioning in patients with depression,^75^ and reduce the need for a trial-and-error approach associated with drug adverse effects that can erode patient trust and hope.

It is also worth noting that personalization of therapy to improve outcomes is not limited to pharmacotherapies. Personalization achieved through the optimization of stimulation targets and parameters of transcranial magnetic stimulation has demonstrated improved efficiency as compared with standard neuromodulation protocols.^80^

## Precision psychiatry findings for a more circuit-driven approach

Given the heterogeneity of depression^81^ and the relatively modest efficacy of existing antidepressants, a move away from simply symptom-based diagnosis is urgently needed. One way this could be achieved is through precision psychiatry, which is an approach to psychiatric treatment that is based on understanding the neurobiological mechanisms that cause symptoms so that treatment can be tailored precisely to those mechanisms.^34^ Concisely, precision psychiatry may be viewed as the right treatment for the right patient,^82^ with the understanding that timing of treatment may also play a key role.

A shift away from the classification structure of DSM-4 to biologically based diagnosis was initiated in 2009 by the United States National Institute of Mental Health as part of a long-term strategic initiative with their Research Domain Criteria Project (RDoC).^28^ ^,^^34^ ^,^^83^ While DSM-5 does incorporate some neuroscience not included in previous versions,^84^ the RDoC aims to develop new ways of classifying mental disorders based on dimensions of observable behavior and neurobiological measures.^85^ ^,^^86^

The dimensions of the RDoC are organized into six superordinate domains of functioning: negative valence, positive valence, cognition, social processes, arousal/regulatory systems, and sensorimotor systems.^85^ Each domain contains several constructs characterized by data from behavior or cognitive function, evidence for a neural circuit, and relevance to psychopathology.^85^ RDoC considers mental disorders from a translational point of view in two steps: in the first step, it determines the primary behavioral functions of the brain and specifies neurobiological systems responsible for these functions; in the second step, psychopathology in terms of dysfunction of different kinds in particular systems is considered from an integrative, multi-systems point of view,^28^ thereby enabling deep phenotyping. Importantly, the RDoC was developed not only to generate the initial constructs framework but also to evolve with scientific progress. Although RDoC is largely theory-based, there is ongoing investigation and validation of the proposed constructs in a data-driven way, as well as in optimizing tools to assess RDoC constructs.^87^ ^,^^88^ While principles of the RDoC have extended into clinical studies, regulatory bodies including the FDA and European Medicines Agency, continue to base study population inclusion criteria on DSM-5 or the International Statistical Classification of Diseases and Related Health Problems diagnostic coding for MDD,^89^ ^,^^90^ presenting possible challenges for this approach.

Technical advances and improved knowledge have provided new insights into the brain circuits that underlie cognitive and emotional functioning.^84^ For example, a possible neural circuit taxonomy has been proposed to address the gap between advances in brain imaging and clinical practice for mental disorders,^84^ in place of a symptom-led taxonomy. Certain dysfunctions in large-scale circuits that control emotional and cognitive functions describe distinct biotypes of depression and anxiety, which may commonly co-occur in individuals.^84^ For example, six neural circuits have been proposed in dysfunctions expressed in depression and anxiety: default mode, salience, negative affect, positive affect (reward), attention, and cognitive control.^84^ Another framework that has been proposed is based on brain lesions networking mapping, where it was shown that functional connectivity between lesion locations and the left dorsolateral prefrontal cortex was strongly associated with depression.^91^ Consequently, this neural circuit is thought to hold promise for precision targeted therapy in individuals with depression.

Other examples of neural circuit-based biotypes may inform pharmacotherapy, the most used treatment for MDD.^84^ In a clinical trial, anterior insula hyperactivation during resting metabolism was identified (via PET scanning) as a differential biomarker of remission for escitalopram.^92^ Similarly, amygdala reactivity to emotional faces was used to identify individuals who are unlikely to respond to particular types of antidepressants in the randomized international Study to Predict Optimized Treatment for Depression (iSPOT-D) clinical trial that combined antidepressant therapy with pre-/post-neuroimaging scans.^93^

Neuroimaging may be used to achieve a more precise diagnosis based on characterizing the underlying neural circuit function, thereby providing the clinician with additional data to inform treatment choices, such as selecting an appropriate pharmacotherapy and limiting side effects.^80^ ^,^^94^ ^,^^95^ In another example, using iSPOT-D data, remission on standard first-line antidepressants depended on pre-treatment connectivity between the posterior cingulate cortex and the anterior cingulate cortex.^96^ Similarly, the comparative effectiveness of existing therapeutics can be explored based on neural circuit changes in response to different compounds. For example, through analyzing data collected from the iSPOT-D trial, it was demonstrated that sertraline responders had higher functional connectivity at baseline between the dorsolateral prefrontal cortex/supramarginal gyrus and supramarginal gyrus/middle temporal gyrus when compared with nonresponders.^97^ The opposite was observed for the venlafaxine-extended release group, where responders had lower functional connectivity in these regions.^97^ Following treatment with sertraline, reduction of connectivity in the precentral and superior temporal gyri was associated with symptom improvement; for the venlafaxine-extended release group, symptom improvement correlated with enhancement of connectivity between the orbitofrontal cortex and subcortical regions.^97^

Resting-state electroencephalography (rsEEG) has also been used to predict the outcome of sertraline versus placebo in a neuroimaging-coupled, placebo-controlled antidepressant study.^98^ EEG may be a more accessible tool for use in clinical practice, even with its relatively reduced spatial and temporal resolution compared with magnetic resonance imaging (MRI).^98^ ^,^^99^ Symptom improvement predicted using the sertraline rsEEG signature was associated with prefrontal neural connectivity and was found to be consistent across different study sites and EEG equipment.^98^

## Examples of drug development techniques that may support precision psychiatry

Many innovative strategies have been used in the development of pharmacological agents some of those used in MDD, which may prove useful for precision psychiatry approaches, are discussed in Table 2.

**Table 2.** Examples of pharmacological development strategies being implemented to meet current unmet needs for patients with depression which may support precision psychiatry

Drug	Mechanism of action	Stage of development	Precision psychiatry strategies	
			**UNMET NEED -> PRECISION PSYCHIATRY STRATEGY**	
			**Too many clinical trial failures due to high placebo response - > Mitigation of placebo response**	
Rel–1017^138^ ^,^^139^	NMDA receptor channel blocker	Phase III (ongoing)	Post hoc analyses to mitigate the implausible positive placebo responses from two high-enrolling sites	
			**Side effects lead to premature discontinuation - > Improving tolerability**	
Lumateperone^140^	Simultaneous modulation of dopamine, serotonin, and glutamate neurotransmission	Approved in adults with bipolar I or bipolar II disorder (completed)	Selection of compounds with no interaction with receptors that contribute to cardiometabolic side effects associated with other antipsychotic medications	
SEP–4199 CR (non-racemic 85:15 ratio of aramisulpride: esamisulpride)^141^ ^,^^142^	Increases potency for serotonin 7 receptors and reduces the level of dopamine D_2_ receptors	Phase III (ongoing)	Use a non-racemic form rather than a racemic form may lower the rate of movement disorder/extrapyramidal symptoms via reduced central D_2_ receptor blockade	
			**Standard trial designs have led to many trial failures -> Innovative trial design**	
Mitizodone phosphate^143^	Selective serotonin partial agonist and reuptake inhibitor	Phase II and Phase III (ongoing)	Adaptive study design to determine optimal dose in Phase II and confirm the dosage for efficacy and safety in Phase III	
			**Many pharmacological agents will fail to be approved for their possible indications - > Developed via proof of mechanism under the “Fast-Fail”**	
Aticaprant^100^	Selective kappa opioid receptor antagonist	Phase III (ongoing)	Evaluated as an adjunctive therapy to an antidepressant in patients who have had an inadequate response to current antidepressant therapy with an SSRI or SNRI	
			**Delayed onset of antidepressant effect - > Improve onset of action**	
SAGE–217 (zuranolone)^144^	Positive allosteric modulator of gamma-aminobutyric acid A receptor	New Drug Application in MDD filed^145^ (completed) Approved in adults for treatment of postpartum depression^146^	Unlike SOC treatment, which can take weeks or months to demonstrate efficacy, zuranolone has a rapid onset of action, potentially reducing negative outcomes such as the likelihood of remission and nonadherence	
Esketamine^12^	NMDA receptor antagonist	Approved for treatment-resistant depression (completed)	Rapid onset of action may be relevant in the acute care of patients with MDD who have active suicide ideation with intent	
			**Clinical trials are not ecologically valid - > Real-world clinical practice**	
Vortioxetine^147^	Inhibitor of serotonin transporter and modulates several serotonin receptor subtypes	Approved in MDD (completed)	Multimodal action demonstrating efficacy across several symptom domains in a real-world heterogeneous population of patients in which >50% had comorbid anxiety, meaning the results are generalizable to a typical MDD population	
			**Depression is a pleiotropic disorder - > Rational combination strategies to target difficult-to-treat symptoms**	
Brexpiprazole^148^	Serotonin–dopamine activity modulator with partial agonism at serotonin 1A and dopamine D_2_ receptors	Approved as adjunctive therapy in MDD^149^ (completed)	Designed for a lower potential for D_2_ receptor agonist-mediated effects (akathisia) and sedation versus other antipsychotics used adjunctively with antidepressants	
Seltorexant^150^	Selective antagonist of the human orexin–2 receptor	Phase III (ongoing)	Developed as an adjunctive treatment for MDD with insomnia symptoms	
Celecoxib^151^	Selective inhibition of cyclooxygenase–2 (responsible for prostaglandin synthesis)	Phase III (ongoing)	Selectively targets key inflammatory pathophysiological pathways and is therefore being examined in a group of patients with depression characterized by the clustering of inflammatory/metabolic dysregulations	
			**Treatment failures are associated with rapid relapse - > Increasing the time before relapse of symptoms**	
Duloxetine^152^	Inhibitor of serotonin and norepinephrine reuptake	Approved for MDD	Inclusion of both an acute (12 week) and continuation (26 weeks) phase to examine the impact of continued treatment with the study drug versus placebo on relapse	
			**Existing antidepressant treatments have resulted in only about one-third of patients improving - > Novel mechanism of action/approaches**	
BI 1569912^153^ ^,^^154^	Negative allosteric modulator of NR2B-containing NMDA receptors	Phase I (completed)	The NR2B subunit is considered key in mediating the efficacy of ketamine. Preclinical studies showed an antidepressant-like effect without induction of tolerance after repeated dosing, modulation of electroencephalogram signature, and favorable psychotomimetic profile compared with (S)-ketamine	
XEN1101 ^155^	KCNQ-selective channel opener	Phase II	Targeting KCNQ2/3 may lead to a change in activation within the bilateral ventral striatum in response to reward as measured by fMRI during an incentive flanker task^156^	
Navacaprant^100^	KOR antagonist	Phase III (ongoing)	Targeting the KOR system may offer therapeutic benefits for neurobehavior disorders^157^	
ALTO–300^158^ ^,^^159^	Melatonergic MT1/MT2 agonist and 5-HT 2C antagonist.	Phase II	Machine learning-driven assessment of baseline electroencephalogram to detect brain biomarker for clinical response ^160^	

Abbreviation: fMRI, functional magnetic resonance imaging; KNCQ, Voltage-gated potassium channels; KOR, kappa opioid receptor; MDD, major depressive disorder; MoA, mechanism of action; MT1, melatonin receptor type 1A; MT2, melatonin receptor type 1B; NMDA, *N*-methyl-D-aspartate; NR2B, N-methyl D-aspartate receptor subtype 2B; SNRI, serotonin-norepinephrine reuptake inhibitors; SOC, standard-of-care; SSRI, selective serotonin reuptake inhibitor.

Notably, the proof-of-mechanism strategy employed for aticaprant, a kappa opioid receptor antagonist^100^ ^,^^101^ may be one of the most useful approaches for the development of psychiatric agents, specifically in the realm of precision psychiatry. Aticaprant was developed based on the Fast-Fail Trials initiative developed by the National Institute of Mental Health.^101^

As many pharmacological agents will fail to be approved for their possible indications, the concept of “Fast-Fail” was developed with the goal of eliminating these agents at earlier, less costly stages of clinical development.^102^ To be developed under “Fast-Fail,” potential agents must meet four requirements: (1) Compelling preclinical research establishing that engaging the target would likely have a therapeutic effect on the brain; (2) Engagement of the target by a compound can be measured in a robust method; (3) The compound specifically engages with the target and preclinical safety data supports human trials; (4) A brain biomarker with a therapeutic potential to serve as the proof-of-mechanism outcome measure for the study.^102^ Although aticaprant was identified for Phase III clinical trial by the “Fast-Fail” trial approach,^100^ there are inherent drawbacks to applying this strategy. Firstly, in psychiatric disorders, there is a limited availability of biomarkers suitable for study outcomes, and not all targets of interest have a robust means of measuring target engagement. It may be possible for the investigational drug to impact other targets and cause clinical changes but not engage the prespecified target. Based on the “Fast-Fail” criteria, this would result in a negative study result, highlighting the need for an established, sufficiently sensitive primary outcome.^102^

Mechanistically driven approaches have been used in other areas of pharmacological development, such as for the development of valbenazine, a reversible vesicular monoamine transporter-2 (VMAT-2) inhibitor used for the treatment of tardive dyskinesia.^103^ ^,^^104^ Tetrabenazine is an approved treatment for chorea associated with Huntington’s disease and has demonstrated improvements in hyperkinetic movement disorders.^105^ ^,^^106^ Valbenazine and tetrabenazine have a common isomer, which was found to be the most potent inhibitor of VMAT-2, supporting the development of this mechanism-based therapeutic.^104^ These developments highlight the potential benefits of mechanistically driven clinical research, which may support precision psychiatry approaches.

## Precision psychiatry to inform phenotypes

There is a need to develop combinatorial diagnostic approaches and tools that can be applied in precision psychiatry to inform phenotypic profiles of patients in clinical settings.^107^ For example, multi-omics and neuroimaging data can be used as biomarkers to achieve a more precise diagnosis that will assist clinicians in offering the right treatment.^94^ ^,^^107^

The use of artificial intelligence methods is still in its infancy in terms of forecasting drug treatments in psychiatry. In time, probabilistic symptom targeting, as well as deep learning algorithms, may be used to predict treatment response, prognosis, diagnosis, and detection of potential biomarkers.^107^ ^,^^108^ For example, a machine learning algorithm using a multidomain data integration model consisting of peripheral blood and cognitive markers was used to predict the diagnosis of bipolar disorder.^109^ Compared with control, a sensitivity of 80% and specificity of 71% was observed for bipolar disorder, suggesting that these blood and cognitive biomarkers could be used by clinicians for diagnosis depending on the clinical situation.^109^ Similarly, a probabilistic graphical model followed by unsupervised machine learning was used to identify specific depressive symptoms and thresholds of improvement that predicted antidepressant response by 4 weeks and the achievement of remission, response, or nonresponse by 8 weeks in 947 patients with depression.^108^ Specific thresholds of change in 4 depressive symptoms, namely depressed mood, feelings of guilt and delusion, work and activities, and psychic anxiety, at 4 weeks predicted the subsequent outcome at 8 weeks to SSRI therapy with an average accuracy of 77%.^108^ In another study, a multisite trial of sertraline versus placebo for adults with MDD was performed using a combination of machine learning with a Personalized Advantage Index (PAI).^110^ The study determined whether individualized treatment recommendations can be generated based on endophenotype profiles coupled with clinical and demographic characteristics.^110^ The study found that a subset of patients with MDD optimally suited to sertraline could be identified based on pre-treatment characteristics, which included higher baseline severity of depressive symptoms, older patients, higher neuroticism, less impairment in cognitive control, and being employed.^110^ Further work is needed, including prospective tests in which the PAI model is built and tested in 2 different samples, but the results of this study demonstrate the potential to use algorithms to predict treatment outcomes. Ultimately, comparative effectiveness trials of relatively comparable treatments or treatment approaches will be a cornerstone for precision psychiatry.

Notably, improved accessibility and increased sharing of health-related data between institutions and sectors for research and clinical uses may further advance the use of artificial intelligence.^111^ Facilitating analysis of the electronic health record with the use of artificial intelligence allows for more personal care by identifying at-risk patients for early intervention or for generating an actionable insight for these patients.^112^

Neuroimaging techniques such as PET and MRI have been used to study the impact of genetic variants on drug target engagement.^113^ A placebo-controlled, crossover study of healthy volunteers and patients with MDD used these neuroimaging techniques to evaluate serotonin transporter occupancy after infusion with citalopram (an SSRI) to assess the impact of *ABCB1* gene variants on drug target engagement in the brain.^113^ Six *ABCB1* single nucleotide polymorphisms were tested, and lower serotonin transporter occupancy was found in *ABCB1* rs2235015 minor allele carriers compared with major allele homozygotes, as well as in men compared with women.^113^ These results highlight the potential of imaging genetics for precision pharmacotherapy in psychiatry.

## Use of pharmacogenomics to target treatment

The first large-scale study to utilize pharmacogenetic (PGx)-guided selection in MDD yielded mixed results.^114^ This was a prospective, double-blind, randomized controlled trial conducted in Spain to assess whether PGx-guided treatment is more effective than unguided treatment in improving drug response and tolerability.^114^ Although no difference in sustained response (primary endpoint) was observed between patients receiving PGx-guided treatment and patients receiving treatment as usual during the study period, the PGx-guided treatment group had a higher responder rate at Week 12. This effect was stronger in patients with 1–3 previously failed psychiatric treatments, with a 2.4-fold increase in the odds of response for these patients. Additionally, PGx-guided treatment resulted in an improved likelihood of achieving better medication tolerability compared with treatment as usual. The results suggest that the use of PGx information to guide treatment adjustments may be justified if traditional first-line treatment fails.^114^

Another multicenter, prospective, double-blind, randomized controlled trial in the USA used pharmacogenetic testing to guide medication management recommendations for depression and anxiety based on gene-drug and drug-drug interactions for over 40 medications used in the treatment of depression and anxiety.^115^ Response and remission rates at Weeks 8 and 12 were significantly higher for patients receiving PGx-guided treatment compared with patients treated with the usual standard of care. There was no statistical difference in adverse drug events between the two groups.^115^ The randomized controlled Precision Medicine in Mental Health Care; PRIME Care trial of 1944 patients with MDD compared treatment guided by pharmacogenomic testing versus usual care.^116^ The PRIME Care study demonstrated that pharmacogenomic testing for drug-gene interactions reduced the prescription of drugs with predicted drug-gene interactions compared with the usual care. However, while remission rates were modestly higher at Weeks 8 and 12 in the pharmacogenomic testing group compared with patients receiving usual care, no advantage was observed at Week 24.^116^

A pharmacogenomic and survival analysis was used to determine suitable antidepressants for the Chinese population.^45^ A total of 610 patient samples were treated with a selection of SSRIs, serotonin norepinephrine reuptake inhibitors (SNRIs), noradrenergic, and specific serotonergic antidepressants (NaSSA) or TCAs.^45^ The study indicated that treatment with SSRIs and SNRIs was more efficacious than with TCAs and NaSSAs in the Chinese population. The study also showed that certain genetic variants were significantly susceptible to a worse response to fluoxetine; these genes were present on the neurotrophin pathway in patients with depression comorbid with anxiety.^45^

Further, a phase 2b trial in participants with treatment-resistant depression utilized the novel genomic biomarker Denovo Genomic Marker 4 (DGM4) to predict the antidepressant response of a novel agent, liafensine. Results of this biomarker-guided study indicated significant improvements in treatment-resistant depression following treatment with liafensine, leading to a Fast Track designation by the FDA.^117^ ^–^^119^

The clinical decision of whether to use pharmacogenomic testing should be guided by a risk–benefit analysis. While the cost of implementing pharmacogenomic testing is likely high, there are potential benefits to the individual patient in providing precise care.^116^

## Challenges to the application of precision psychiatry

While some benefits of precision psychiatry have been outlined above, there are several challenges that may limit clinical translation and utility at this time. The majority of clinical trials recruit patients with mild and moderate severities, and generalization in real-life practice cannot be made to patients with severe disease.^79^

Disagreement exists around the validity of grouping depression into specific subtypes based on symptoms and the presence of specific endophenotypes.^120^ There are variations in the methods of data collection and technical complexity required to process and analyze multi-omics data from large datasets and/or artificial intelligence.^79^

The cost-effectiveness of some of the techniques used in precision psychiatry is still not well known, nor is the cost of appropriate training of healthcare staff in these different techniques.^79^ Ethical concerns, such as protecting the privacy and security of data and patient stratification (risk of discrimination against patients in less privileged groups), also exist.^79^ Additional studies related to the cost-effectiveness of precision psychiatry are warranted to ensure improved treatment approaches are accessible to all patients.

Other challenges faced in the precision psychiatry field are the lack of validated biomarkers that can serve as viable targets for precise therapeutics,^96^ as well as a lack of comparative effectiveness studies. Guidance on personalized treatments, including the type and length of treatments, and more studies using extended follow-up of individuals treated for depression are needed. The results of a meta-analysis indicated that maintenance therapy should be continued for at least 6 months after remission.^121^ This meta-analysis also suggested that continuing antidepressants for another year led to lower relapse rates in patients with MDD, and flexible dose adjustment based on symptoms could help prevent relapse.^121^

## Precision psychiatry for children, adolescents, and specific populations of adults

In the USA, 17% of adolescents had at least one major depressive episode in 2020.^122^ The risk of experiencing MDD is highest during adolescence and is associated with adverse consequences persisting well into adulthood.^123^ During adolescence, the neurocircuitry involved in depression is still developing, and it is crucial that mental health problems are identified and treatment is initiated during this period.^123^ ^,^^124^

Due to the substantial side effects associated with psychiatric medications, clinicians often initiate pharmacotherapy at low doses in children and adolescents and slowly titrate the dose. This may increase the risk of under-treatment and may lead to a medication change due to the lack of treatment response.^125^

An increase in the data available for pediatricians to augment existing treatment guidelines^126^ would be of great benefit. Much of the work in pharmacogenomic testing has been conducted in adults, though there has recently been an increase in studies applying these tests as a predictor of treatment response and medication tolerability in pediatric patients.^125^ The rates of placebo response are higher in children and adolescents than in adults, exacerbating the difficulties in establishing efficacious treatments.^62^ ^,^^127^

Digital phenotyping refers to the “moment-by-moment quantification of the individual-level human phenotype in situ using data from smartphones and other personal digital devices.”^128^ This field may be of particular relevance to child and adolescent psychiatry.^124^ An ongoing study, Texas Resilience in Adolescent Development, follows participants 10–24 years of age at risk for depression. The aim of the study is to uncover the sociodemographic, lifestyle, clinical, psychological, and neurobiological factors that contribute to mood disorder onset, recurrence, progression, and differential treatment response.^129^

Other populations of adults that could benefit from precision psychiatry are older adults and those with BD, as there are clinical challenges in differentiating BD from non-bipolar depression, which often leads to delays in diagnosis and accurate treatment. A more targeted treatment could also be used for pregnant people with depression during the perinatal or postpartum period. Perinatal depression (PND) is heterogeneous as there are likely multiple contributing etiologies and neurohormonal responses.^130^ ^,^^131^ Neurosteroid targets that attend to the neurohormonal context of depression in the postpartum period have recently been approved by the FDA.^131^ ^–^^133^ Determining the biological features responsible for PND could shed light on how precision psychiatry may be used to tailor treatment options.^130^

## Suggested approaches for future clinical studies

Overall, a more targeted approach to treatment using precision psychiatry may offer future benefits to patients. Continued use of the National Institute of Mental Health Fast-Fail Trials may serve as a starting point. As this initiative is based on preclinical research establishing that the engaging target could have a therapeutic effect on the brain and using brain biomarkers to serve as the proof of mechanism outcome measure for the study,^102^ and therefore may offer an improved strategy for the development of psychiatric agents.

Additionally, increased use of human induced pluripotent stem cells (iPSCs) in psychiatric research may also help drive precision psychiatry efforts. IPSCs offer a reproducible method for modeling human diseases,^134^ and in some conditions, iPSCs models have demonstrated aspects of the intended disease compared with controls.^135^ ^,^^136^ Future research may benefit from further use of iPSCs, including the use of brain organoids,^137^ and in disease processes where murine and human physiology vary.^136^

## Conclusions

An unmet need exists in MDD to develop diagnostic tools and more targeted therapy using precision psychiatry-based approaches so that the right treatments can be matched to the right patients. Identification of clinical biomarkers may allow for a more precise approach to treatment, in which specific disease mechanisms (which may sometimes be shared across multiple disorders) are targeted. PGx testing, neuroimaging, and machine learning approaches have been used with some success in trial settings, and some neural circuit biotypes associated with MDD have been identified. The core challenge remains as targeted receptor physiology is only part of the complex dysfunction due to the wide distribution of many receptors. Further advances in precision psychiatry pharmacotherapy may hinge on the spatial identification of selective subclasses of receptors. Still, clinical biomarkers that can effectively predict response to treatment based on individual phenotypes are needed. Personalized treatments could improve remission rates and reduce the risk of relapse, leading to overall better functioning in patients with depression.
